# A newly designed uncovered biliary stent for palliation of malignant obstruction: results of a prospective study

**DOI:** 10.1186/s12876-020-01325-9

**Published:** 2020-06-10

**Authors:** Christopher Lawrence, Jose Nieto, Willis G. Parsons, André Roy, Nalini M. Guda, Stephen E. Steinberg, Muhammad K. Hasan, Juan Carlos Bucobo, Satish Nagula, Nicholas D. Dey, Jonathan M. Buscaglia, On behalf of the Study Investigators

**Affiliations:** 1 Charleston Gastroenterology Specialists, Charleston, SC USA; 2 Borland-Groover Clinic, Jacksonville, FL USA; 3 Northwest Community Healthcare, Arlington Heights, IL USA; 4 Centre Hospitalier de l’Université de Montréal, Montréal, QC Canada; 5 Aurora Saint Luke’s Medical Center, Milwaukee, WI USA; 6 The Pancreas and Biliary Center of South Florida, Boca Raton, FL USA; 7 Florida Hospital, Orlando, FL USA; 8 Division of Gastroenterology and Hepatology, Stony Brook University Hospital, 101 Nicolls Road, HSC Building, 17th floor, Room 063, Stony Brook, New York, Stony Brook, NY 11794 USA; 9 Cook Research Incorporated, West Lafayette, IN USA

**Keywords:** Self expandable metallic stent, Biliary tract neoplasms, Palliative medicine, Stents, Prosthesis design

## Abstract

**Background:**

Biliary decompression can reduce symptoms and improve quality of life in patients with malignant biliary obstruction. Endoscopically placed stents have become the standard of care for biliary drainage with the aim of improving hepatic function, relieving jaundice, and reducing adverse effects of obstruction. The purpose of this study was to evaluate the performance characteristics of a newly-designed, uncovered metal biliary stent for the palliation of malignant biliary obstruction.

**Methods:**

This post-market, prospective study included patients with biliary obstruction due to a malignant neoplasm treated with a single-type, commercially available uncovered self-expanding metal stent (SEMS). Stents were placed as clinically indicated for palliation of jaundice and to potentially facilitate neo-adjuvant chemotherapy. The main outcome measure was freedom from recurrent biliary obstruction (within the stent) requiring re-intervention within 1, 3, and 6 months of stent insertion. Secondary outcome measures included device-related adverse events and technical success of stent deployment.

**Results:**

SEMS were placed in 113 patients (73 men; mean age, 69); a single stent was inserted in 106 patients, and 2 stents were placed in 7 patients. Forty-eight patients survived and/or completed the 6 month study protocol. Freedom from symptomatic recurrent biliary obstruction requiring re-intervention was achieved in 108 of 113 patients (95.6, 95%CI = 90.0–98.6%) at study exit for each patient. Per interval analysis yielded the absence of recurrent biliary obstruction in 99.0% of patients at 1 month (*n* = 99; 95%CI = 97.0–100%), 96.6% of patients at 3 months (*n* = 77; 95%CI = 92.7–100%), and 93.3% of patients at 6 months (*n* = 48; 95%CI = 86.8–99.9%). In total, only 5 patients (4.4%) were considered failures of the primary endpoint. Most of these failures (4/5) were due to stent occlusion from tumor ingrowth or overgrowth. Overall technical success rate of stent deployment was 99.2%. There were 2 cases of stent-related adverse events (1.8%). There were no cases of post-procedure stent migration, stent-related perforation, or stent-related deaths.

**Conclusions:**

This newly designed and marketed biliary SEMS system appears to be effective at relieving biliary obstruction and preventing re-intervention within 6 months of insertion in the overwhelming majority of patients. The device has an excellent safety profile, and associated high technical success rate during deployment.

**Trial registration:**

The study was registered on clinicaltrials.gov on 14 October 2013 and the study registration number is NCT01962168. University of Massachusetts Medical School did not participate in the study.

## Background

Biliary decompression can reduce symptoms and improve quality of life in patients with malignant biliary obstruction [1–4]. Resolving the obstruction may also allow for palliative chemotherapy [3]. Due to their minimal invasiveness compared to percutaneous drainage, endoscopically placed stents have become the standard of care for non-surgical biliary drainage [1, 5]. The goal of stent placement is to improve hepatic function, normalize bilirubin levels, relieve symptoms of jaundice, and reduce other adverse effects of obstruction [1, 3, 6, 7].

There are a variety of stents available and the choice of device is often based on several factors, including certainty of malignancy diagnosis, potential need for removal, expected length of survival, location of obstruction, and cost-effectiveness [2, 3, 7–9]. Plastic stents are less expensive than self-expanding metal stents (SEMS) and can be removed easily, if necessary [7]. However, plastic stents are known to have a high rate of occlusion and the risk of occlusion increases over time [1, 3, 5, 9–12]. While SEMS are more expensive than plastic stents, long stent patency and reduced need for re-intervention makes SEMS a more cost-effective option [1, 5, 10, 12, 13].

**Fig. 1:**
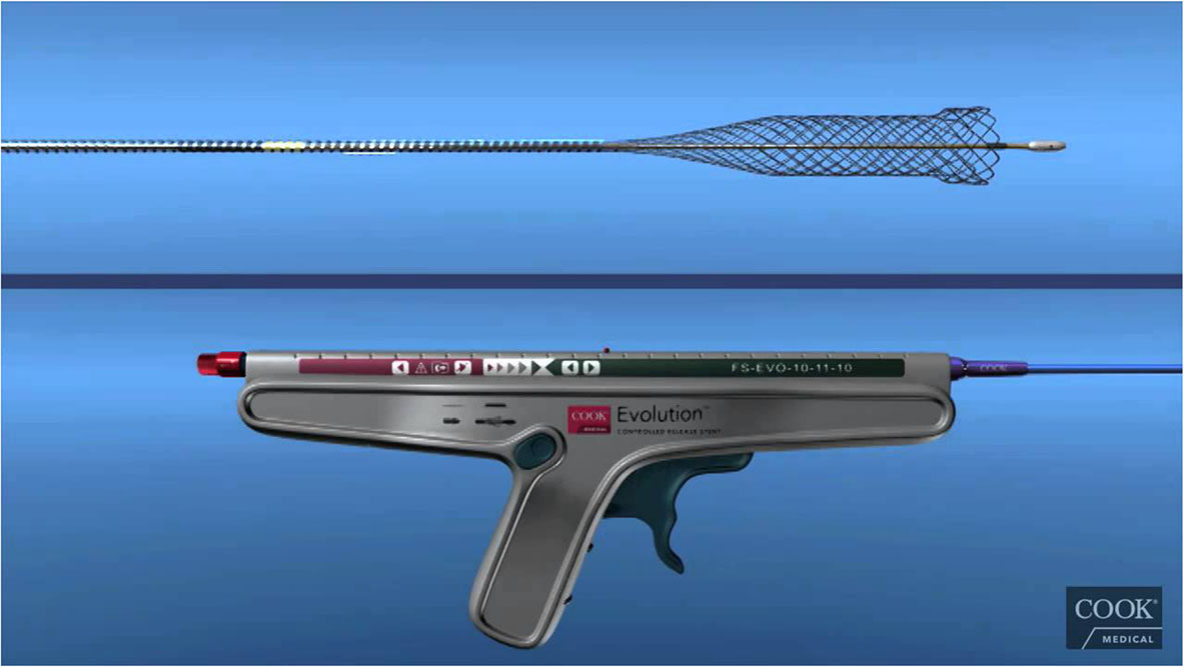
The Evolution® Biliary Stent System – Uncovered. Image provided with written permission for use from Cook Medical Inc.

The aim of this study was to evaluate the performance of a newly-marketed, uncovered biliary stent system (Evolution® Biliary Stent System – Uncovered; Cook Ireland Ltd., Limerick, Ireland) for the palliation of malignant biliary obstruction (Fig. 1). The primary outcome measure was freedom from symptomatic recurrent biliary obstruction requiring another intervention. Secondary outcome measures included technical success of attempted stent placement, ease-of-use of the device, and device-related adverse events.

## Methods

### Patients

Patients who underwent biliary stent placement for the treatment of biopsy-proven, malignant biliary tree obstruction were prospectively enrolled at 8 medical centers (7 US, 1 Canada) between December 2013 and October 2014. This trial was registered with ClinicalTrials.gov under the following trial number: NCT01962168. University of Massachusetts Medical School did not participate in the study. Patients who were 18 years or older, with the ability to provide informed consent, as well as comply with the post-procedural follow-up schedule, were eligible for inclusion in the study. SEMS were placed as clinically indicated, for the purposes of palliation of jaundice and/or to facilitate expected neo-adjuvant chemotherapy.

### Ethics approval

The study was approved by the institutional review boards (IRB) at each of the respective medical centers (see ‘Ethics Approval and Consent to Participate’ section below in the ‘Declaration’ for list of individual sites). All patients provided informed consent prior to participation in the study. This study was sponsored by Cook Endoscopy.

### Uncovered SEMS

The uncovered SEMS (Cook Evolution® Biliary Stent – Uncovered; Cook Ireland Ltd., Limerick, Ireland) used in this study is constructed of a single, woven, nitinol wire with a radiopaque core. The stent has flanged ends to mitigate migration. The stent is available in 8 or 10 mm diameters and 4, 6, 8, and 10 cm lengths and contains 4 radiopaque markers on the delivery system to assist in deployment under fluoroscopic guidance. The stent is mounted on an inner catheter, which accepts a 0.035 in. wire guide and is restrained from expansion by an outer catheter. The stent is deployed using a controlled-release, trigger-driven delivery system. This system allows for recapturing of the SEMS prior to complete deployment if repositioning is required.

### Stent placement

The placement of the SEMS was performed by experienced endoscopists during endoscopic retrograde cholangiopancreatography (ERCP) with patients under both moderate and deep sedation at the various 8 participating centers. Table 1 outlines the number of patients enrolled at each of the 8 different centers.

**Table 1:** Patient enrollment at each of the participating centers (*N* = 113)

Stony Brook University Hospital	Stony Brook, NY USA	15
Mobile Infirmary Medical Center	Mobile, AL USA	4
Aurora St. Luke’s Medical Center	Milwaukee, WI USA	13
Florida Hospital Orlando	Orlando, FL USA	8
Borland-Groover Clinic	Jacksonville, FL USA	20
Northwest Community Hospital	Arlington Heights, IL USA	20
Centre Hospitalier de l’Université de Montréal	Montréal, QC Canada	20
Pancreas and Biliary Center of South Florida	Boca Raton, FL USA	13

### Follow-up assessment

Follow-up assessments were conducted at 30 days (1 month), 3 months, and 6 months post-procedure for symptoms of recurrent biliary obstruction, additional hospitalizations or procedures, and adverse events. Assessments were conducted by telephone or, if preferred by the physician and/or patient, by clinical examination.

### Definition of endpoints

The primary endpoint was clinical success, defined as freedom from symptomatic recurrent biliary obstruction (within the stent) requiring re-intervention. Clinical success was assessed for each patient at the time of study exit. Study exit may be due to completion of 6 month follow-up protocol, withdrawal from the study, patient lost to follow-up, symptoms of obstruction requiring re-intervention, stent removal due to reasons other than obstruction, or patient death. Clinical failure occurred when the patient presented with symptoms of biliary obstruction that the physician considered significant enough to warrant re-intervention. If a patient refused re-intervention, the case was conservatively considered as a clinical failure. If re-intervention was performed, confirmation of complete or partial occlusion of the biliary stent was needed to be considered a failure of the primary endpoint. Cases in which the stent was found to be patent (e.g., obstruction occurred distal or proximal to the stent) were not classified as clinical failures. If a patient died without a known recurrence, they were not considered a failure of the primary endpoint.

Secondary endpoints included: 1) ease-of-use, based on physician assessment of the device after each deployment; 2) incidence of device-related adverse events, including stent migration, stent-related perforation, and stent-related death; 3) technical success, defined as successful delivery and placement of a stent at its intended location, assessed using fluoroscopy at the end of the stent placement procedure. Positional adjustments to the stent during the procedure were not considered technical failures.

### Statistical analysis

A minimum sample size of 112 was calculated based on the rate of freedom from symptomatic biliary obstruction requiring re-intervention for a similar uncovered biliary stents. Data were collected and managed by a centralized data coordinating center. Statistical analyses were performed by a biomedical statistician using Statistical Analysis Software (SAS) for Windows (release 9.3 or higher). Continuous variables are reported as mean (standard deviation) unless otherwise noted and categorical variables are reported as percentages. A Clopper-Pearson exact 95% binomial confidence interval was calculated for the primary study endpoint of freedom from recurrent biliary obstruction requiring re-intervention, based on clinical success assessment for each patient at the time of study exit. Additionally, a Kaplan-Meier analysis was performed to estimate clinical success during follow-up at 30 days, 3 months, and 6 months post-procedure. The analysis accounts for patients who exited the study without a documented clinical failure and who were censored at the time of study exit.

## Results

A total of 113 patients (73 [64.6%] male; mean age, 69 ± 12 years) with malignant biliary obstruction were enrolled. Thirty patients (26.5%) underwent pre-procedural or adjunctive tumor reduction therapy with systemic chemotherapy. A previous biliary sphincterotomy was noted in 28 patients (24.8%), and sphincterotomy was performed at the time of SEMS placement in 75 patients (66.4%). Prior attempts at biliary decompression of the stricture were noted in 54 patients. This included 40 patients (35.4%) with plastic biliary stents, 5 patients (4.4%) with percutaneous biliary drain placement, and 9 patients (8.0%) with balloon dilation of the stricture. The majority of patients (79, or 69.9%) had pancreatic cancer, and advanced stage disease (T3N1) at initial presentation (Table 2).

**Table 2:** Patient demographics, cancer etiology, and stricture morphology (*N* = 113)

Characteristic	N (%)
Age, years	
Mean ± standard deviation (range)	68.8 ± 12.2 (33–93)
Gender	
Male	73 (64.6)
Female	40 (35.4)
Race/Ethnicity	
White	97 (85.8)
Black or African American	9 (8.0)
Hispanic or Latino	6 (5.3)
Asian	1 (0.9)
Tumor type	
Pancreatic cancer	79 (69.9)
Cholangiocarcinoma	13 (11.5)
Gallbladder cancer	2 (1.8)
Gastric cancer	2 (1.8)
Ampullary carcinoma	1 (0.9)
Duodenal carcinoma	1 (0.9)
Other^a^	15 (13.3)
Total bilirubin (mg/dL)^b^	
Mean ± standard deviation (N, range)	8.3 ± 7.8 (102, 0.1–37.5)
Stricture morphology	
Stricture location	
Extrahepatic	104 (92.0)
Intrahepatic	4 (3.5)
Both intrahepatic and extrahepatic	5 (4.4)
Stricture length (cm)	
Mean ± standard deviation (range)	2.3 ± 1.2 (0.8–8.0)
TNM Stage^c^	
T1	0/105 (0.0)
T2	10/105 (9.5)
T3	64/105 (61.0)
T4	31/105 (29.5)
N0	33/103 (32.0)
N1	54/103 (52.4)
N2	10/103 (9.7)
N3	6/103 (5.8)
M0	57/100 (57.0)
M1	43/100 (43.0)

^a^ The majority of other tumor types are metastases

^b^ Collection of total bilirubin was not standard of care at all medical centers; therefore, data were not collected for 11 patients

^C^ T stage information not available for 8 patients, N stage not available for 10 patients, M state not available for 13 patients

Most strictures (92, 81.4%) were completely or partially localized to the common bile duct; 96 stents (80.0%) did not extend above the biliary confluence (Table 2). The majority of stents (100, 83.3%) crossed the papilla. A single SEMS was placed in 106 patients (93.8%); 2 SEMS were placed in 7 patients (6.2%). The most common stent diameter and length used in this study were 10 mm (85.8%) and 60 mm (59.2%), respectively.

**Fig. 2:**
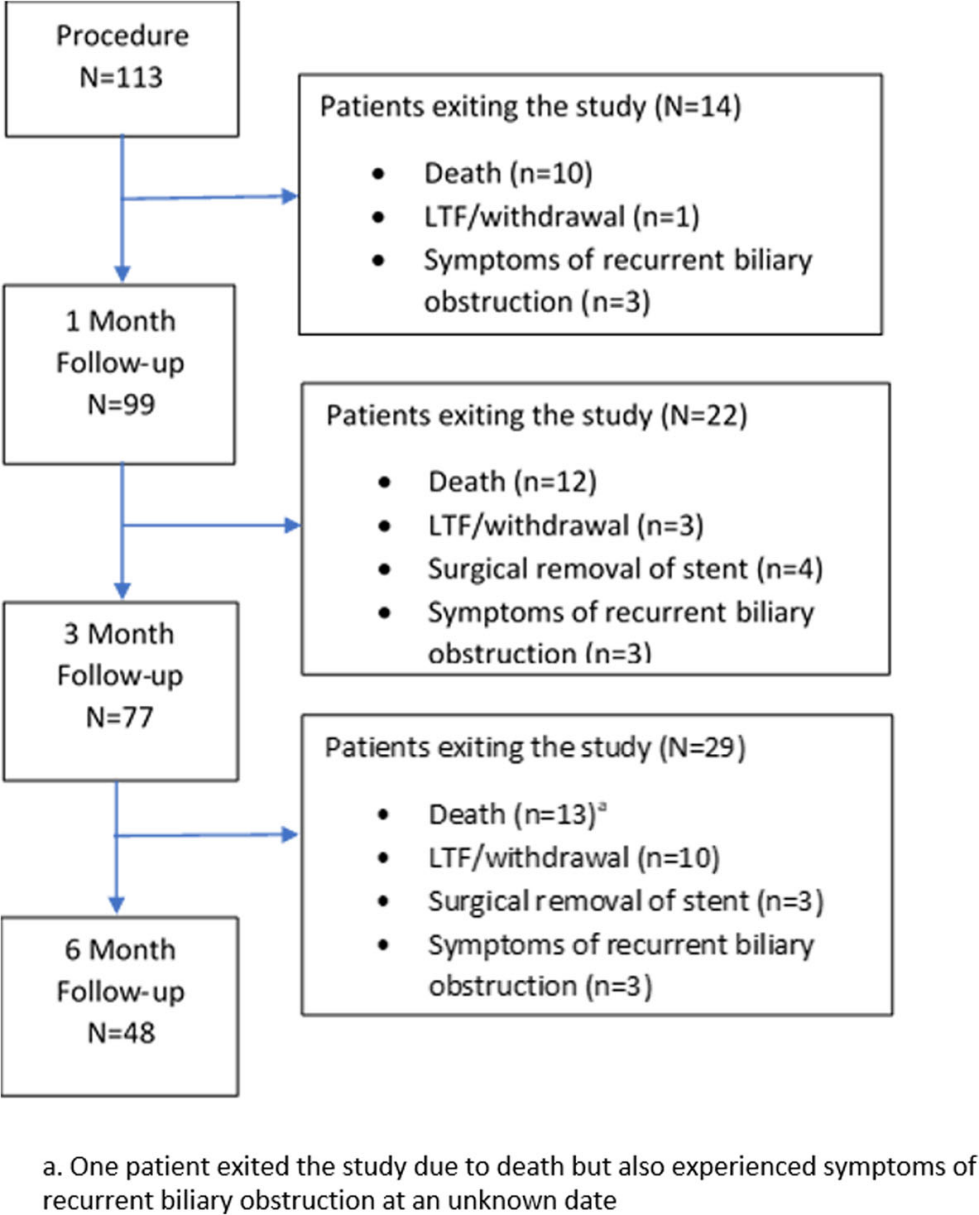
Patient flowchart of enrollment, retention, and clinical follow-up

Figure 2 represents a summary of patient enrollment and retention for procedure and clinical follow-up. Over the course of the study, 13 patients (11.5%) were lost to follow-up and 1 patient (0.9%) voluntarily withdrew from the study. A total of 48 patients (42.5%) had a follow-up assessment at 6 months. Throughout the course of the study, a total of 10 patients (9 + 1, or total of 8.8%) developed symptomatic recurrent biliary obstruction. In addition to the 9 patients, one patient exited the study due to death but developed biliary obstruction prior to dying. Of the 9 patients with recurrent biliary obstruction, only 5 patients underwent re-intervention (see *Clinical Success* below).

### Clinical success

**Fig. 3:**
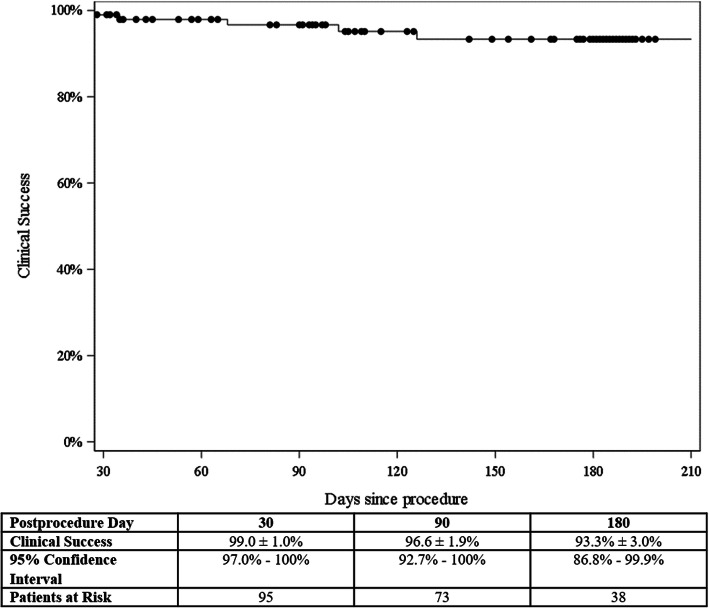
Clinical Success - Freedom from symptomatic recurrent biliary obstruction (within the stent) requiring re-intervention

The primary study endpoint, clinical success, defined as freedom from symptomatic recurrent biliary obstruction (within the stent) requiring re-intervention, was achieved in 108 of 113 patients (95.6, 95%CI = 90.0–98.6%) at study exit for each patient. Kaplan-Meier analysis was performed to assess clinical success during follow-up (Fig. 3), which indicated freedom from obstruction requiring re-intervention in 99.0% ± 1.0% at 1 month (95%CI = 97.0–100%; *n* = 99 remaining patients), 96.6% ± 1.9% at 3 months (95%CI = 92.7–100%; *n* = 77 remaining patients), and 93.3% ± 3.0% at 6 months (95%CI = 86.8–99.9%; *n* = 48 remaining patients). As noted, 10 patients presented with symptoms of recurrent biliary obstruction over the course of the study; however, only 5 cases (4.4%) were considered failures of the primary endpoint. The majority of these failures (4/5) were due to stent occlusion from tumor ingrowth and/or overgrowth. The other 5 cases not considered failures were due to obstruction either proximal (*n* = 4) or distal (*n* = 1) to the existing metal stent, but not involving the actual stent. This was due to biliary stones or sludge, food debris, or tumor in some cases.

### Technical success and device performance

Technical success was achieved in 112/113 patients (99.1%, CI 95% = 95.1–100.0%) and in 119/120 stent placements (99.2%). The single failure was due to difficulty removing the delivery system from a tight stricture, resulting in distal stent migration (1 cm) during the procedure. As a result, a non-study stent was placed inside the SEMS to ensure adequate stent coverage in the duct.

Ease of stent deployment was rated as ‘easy’ (53/120) or ‘very easy’ (57/120) in the majority of cases (91.7%). The deployment of 3 stents, placed in 2 patients, was rated as ‘difficult’ (1/120, 0.8%) or ‘very difficult’ (2/120, 1.7%). One of these patients had a moderately tortuous stricture at the confluence of the left and right main intrahepatic ducts, and 2 stents were placed. In all 3 cases deployment was successful. In addition, fluoroscopic visualization of the SEMS was rated as ‘easy’ (*n* = 25) or ‘very easy’ (*n* = 87) for 112 stents (93.3%). Stent recapture was required during 6 deployments (5.0, 95%CI = 1.9–10.6%); 1 stent was recaptured twice. Recapture was rated as ‘very easy’ in all cases and the stents were successfully deployed.

### Adverse events and deaths

Only two patients (1.8, 95%CI = 0.4–10.1%) had device-related adverse events, which were classified by the physician as probably or definitely related to the device deployment. In both cases, the device-related adverse event resulted in biliary obstruction requiring re-intervention before 6 months’ time. Both patients were successfully treated with the placement of a non-study stent. The remaining adverse events were not stent-related; and they were categorized as gastrointestinal (GI) in 18 patients, cardiovascular in 3 patients, and miscellaneous in 32 patients. In addition, 7 patients underwent surgical removal of the study stent via pancreatoduodenectomy. There were no cases of post-procedure stent migration or stent-related perforation. There were a total of 38 deaths over the course of the study. None of these deaths were stent-related.

## Discussion

As we are faced with the rising incidence of pancreatic cancer and other malignancies of the upper gastrointestinal tract, the role of non-surgical endoscopic techniques for managing pancreatico-biliary disorders is expanding. As a result, available technologies which enable the endoscopist to intervene in this setting are also growing. The growth in industry and technology, however, does not always parallel the availability of appropriately-designed scientific studies of new devices used in critical patient care settings. In this study, we prospectively evaluated the basic performance characteristics and safety of a newly-designed uncovered biliary SEMS used in the palliation of malignant biliary obstruction. We not only evaluated device-specific endpoints, but we critically evaluated patient-specific outcomes with recurrent biliary obstruction mandating re-intervention as our primary endpoint.

It has long been known that SEMS provide more optimal patency than plastic biliary stents. A recent meta-analysis performed by Sawas and colleagues [10] on the comparison of plastic versus metal biliary stents found a 30-day occlusion rate of 3% for SEMS, and a long-term occlusion rate of 27% for SEMS. In the present study, the overall stent occlusion rate at 6 months was 4.4%. Tumor ingrowth was the cause of occlusion in nearly all of these patients. This is not unexpected when utilizing uncovered SEMS that have open interstices. Furthermore, there were no cases of post-procedure stent migration reported, likely related to the open mesh design enabling the stent to embed itself into the bile duct wall.

The technical success rate of 99.2% achieved in this study is comparable to that observed with other SEMS. Meta-analyses investigating other types of SEMS found an overall technical success rate ranging from 94.6 to 98.8%, [1, 10, 14], and 88.3% with plastic stents [1]. The higher technical success rate of SEMS is likely due to their expandability, facilitating placement in tight strictures while using endoscopes with relatively small working channels [7]. Here, of the 23 stents (19.2%) placed in strictures rated as ‘moderately’ or ‘severely tortuous’ by the treating endoscopists, only one stent was unsuccessfully deployed. This single failure was due to the removal of the delivery system from a tight stricture, resulting in distal stent migration during the procedure.

Overall, ease-of-use was rated extremely favorably by the physicians who performed the procedures in this study. We suspect that much of this positive rating is related to the lack of stent foreshortening while using this device. In general, the low foreshortening rate associated with uncovered SEMS is suggested to make deployment easier compared to covered stents [9]. Dumonceau and colleagues [7] suggest that SEMS with a low foreshortening ratio are superior in cases of long, tight strictures; but they may be associated with a jerky deployment. Deployment with the current device appeared devoid of erratic or uncontrolled release from the delivery system, likely because of its incremental trigger-release system which allows a smoother exit from the catheter.

Despite some of these favorable outcomes noted in our study, it is not without its limitations. Studying patients with advanced malignancies of the upper gastrointestinal tract is frequently challenged by the fact that patients often succumb to their disease process prior to reaching the desired endpoints of the study. This is clearly the case here, as approximately one-third of the patients died prior to reaching the 6 month post-stent follow-up analysis. Most of these patients (81.6%) died of their underlying malignancy. When coupled with a relatively high rate of patients lost to follow-up (14/113, or 12.4%), the number of patients available for analysis is limited, which may reduce the accuracy of the endpoint estimate. Furthermore, additional clinical characteristics of the enrolled patients, such as performance status, comorbidity indices, and duration of stent/drain placement prior to SEMS, were not collected. These important parameters may have an effect on procedure-related complications, likelihood of palliation of jaundice, and overall survival; which, in turn, may affect recurrent obstruction and the need for re-intervention.

## Conclusions

In summary, the recently designed and marketed biliary SEMS system (Cook Evolution® Biliary Stent – Uncovered; Cook Ireland Ltd., Limerick, Ireland) appears to be effective at relieving biliary obstruction and preventing re-intervention within 6 months of insertion in the overwhelming majority of patients. The device has an excellent safety profile, and possess a high technical success rate during deployment in a variety of types of biliary strictures.

## Data Availability

Data underlying the results reported in this article are available upon reasonable request from the corresponding author, immediately after publication and ending 5 years after publication, subject to review and approval by the study sponsor. Interested researchers may review the “Cook Research Incorporated Policy on Access to Clinical Study Data” at https://www.cookresearchinc.com/extranet/data-access.html and submit a complete research proposal to request data access.

## Abbreviations

CI: Confidence interval
ERCP: Endoscopic retrograde cholangiopancreatography
GI: Gastrointestinal
SEMS: Self-expanding metal stent
